# Estimating the inbreeding level and genetic relatedness in an isolated population of critically endangered Sichuan taimen (*Hucho Bleekeri*) using genome‐wide SNP markers

**DOI:** 10.1002/ece3.5994

**Published:** 2020-01-14

**Authors:** Yongquan Zhang, Peixian Luan, Guangming Ren, Guo Hu, Jiasheng Yin

**Affiliations:** ^1^ Heilongjiang River Fisheries Research Institute Chinese Academy of Fishery Sciences Harbin China

**Keywords:** conservation genetics, genomic inbreeding, genomic relatedness, *Hucho bleekeri*

## Abstract

Sichuan taimen (*Hucho bleekeri*) is critically endangered fish listed in The Red List of Threatened Species compiled by the International Union for Conservation of Nature (IUCN). Specific locus amplified fragment sequencing (SLAF‐seq)‐based genotyping was performed for Sichuan taimen with 43 yearling individuals from three locations in Taibai River (a tributary of Yangtze River) that has been sequestered from its access to the ocean for more than 30 years since late 1980s. Applying the inbreeding level and genetic relatedness estimation using 15,396 genome‐wide SNP markers, we found that the inbreeding level of this whole isolated population was at a low level (2.6 × 10^−3^ ± 0.079), and the means of coancestry coefficients within and between the three sampling locations were all very low (close to 0), too. Genomic differentiation was negatively correlated with the geographical distances between the sampling locations (*p* < .001), and the 43 individuals could be considered as genetically independent two groups. The low levels of genomic inbreeding and relatedness indicated a relatively large number of sexually mature individuals were involved in reproduction in Taibai River. This study suggested a genomic‐relatedness‐guided breeding and conservation strategy for wild fish species without pedigree information records.

## Graphical abstract

Specific locus amplified fragment sequencing (SLAF‐seq)‐based genotyping was performed for Sichuan taimen with 43 yearling individuals from three locations in Taibai River (a tributary of Yangtze River) that has been sequestered from its access to the ocean for more than 30 years since late 1980s. Applying the inbreeding level and genetic relatedness estimation using 15,396 genome‐wide SNP markers, we found that the inbreeding level of this whole isolated population was at a low level (=2.6 × 10^−3^ ± 0.079), and the means of coancestry coefficients within and between the three sampling locations were all very low (close to 0), too. Genomic differentiation was negatively correlated with the geographical distances between the sampling locations (*p* < 0.001), and the 43 individuals could be considered as genetically independent two groups.

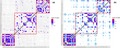

## 1 INTRODUCTION

Sichuan taimen (*Hucho bleekeri*) is a large and fierce carnivorous fish species, with a maximum length of 2 m (Hu, Wang, Cao, & Xiong, 2008). It lives at the top of the food chain and has important value for maintaining the ecological balance of the habitat waters. Some biogeographical scholars have speculated that the *H. bleekeri* is a remnant fish that invaded from the north to the south during the Quaternary glacial period and has important scientific value in many aspects such as animal geography, paleoecology, fish system, and climate change (Song, 2012). At present, the cold‐water fish farming industry has realized commercial farming of *Hucho taimen* in China (Xu et al., 2007). *Hucho bleekeri*, as an important native germplasm resource of *Hucho*, has huge market development potential in aquaculture in China.

*Hucho bleekeri*, a Chinese national secondary protected animal, is currently listed as a critically endangered species in The Red List of Threatened Species compiled by the International Union for Conservation of Nature (IUCN; Hu et al., 2008). *Hucho bleekeri* was originally distributed only in the upper tributaries of Yangtze River with a distribution of about 5,000 km^2^ recorded in 1960s, including the upper reaches of the Min and Qingyi rivers in Sichuan Province, the upper and middle reaches of the Dadu River in Sichuan and Qinghai provinces, and the Xushui and Taibai rivers located in the upper reaches of the Han River in Shaanxi Province, China. The habitats of this fish have been drastically reduced to <100 km^2^ due to the fragmentation of habitats caused by hydropower development, industrial pollution, and illegal fishing, and there are only a few isolated populations in the wild in recent years (Song, 2012). The number of mature individuals of the species is thought to be ranged from 2,000 to 2,500 based on expert judgement, and the wild populations are estimated to be continuously rapidly decreasing and are expected to lose more than 20% adult individuals over the next two generation (Song, 2012). If humans do not interfere with the process of population decline, this species is very likely to fall into the extinction vortex and eventually becomes extinct.

*Hucho bleekeri* is one of the most threatened fish species, and understanding genetic diversity including inbreeding levels and genetic relatedness in the fragmented populations has important practical significance for the genetic management of ex situ conservation, proliferation, and reintroduction to original habitats of this species (Frankham et al., 2017). However, for many years, it has been difficult for researchers to collect *H. bleekeri* sample in the wild. Moreover, these wild individuals do not have pedigree records and cannot be traced more deeply. As a result, we have little knowledge of the genetic structure of this species in the wild (Qi, Chao, Yang, Shen, Tang, 2009; Shen, Tang, & Li, 2006; Wang et al., 2011). Exhilaratingly, in 2012, a population with dozens of adult fish was discovered in Taibai River, Shaanxi Province, giving researchers the opportunity to analyze the genetic structure (Du et al., 2014). In previous studies using microsatellites and mitochondria markers, no evidence of inbreeding was detected in this population and the genetic structure estimation suggested that the wild *H. bleekeri* population in Taibai River expanded a long time ago, but had suffered great losses in recent years (Wang et al., 2016, 2015; Zhang, Wei, Du, & Li, 2016).

Microsatellite‐based research helps us learn the genetic information of this population, but the density is as low as a dozen microsatellite markers, and this small number markers cannot be representative of the genome structural features, making it impossible for researchers to accurately estimate the inbreeding level and genetic relatedness parameters. Traditional methods for accurate estimates of the above parameters require pedigree information which are unavailable for wild fish populations such as *H. bleekeri* (Falconer & Mackay, 1996). In recent decades, with the widely popularization of high‐throughput sequencing technologies, many statistical methods have been proposed to estimate inbreeding and genetic parameters using genome‐wide SNP markers (Da, Wang, Wang, & Hu, 2014; Goddard & Hayes, 2011; Meuwissen, Hayes, & Goddard, 2001; VanRaden, 2008). However, the estimates of the inbreeding level and genetic relatedness using genome‐wide SNP markers have been unavailable in wild *H. bleekeri* populations.

In the present study, specific locus amplified fragment sequencing (SLAF‐seq)‐based genotyping (Sun et al., 2013) was performed for 43 individuals captured from three locations in the Taibai River, then, the inbreeding level and genetic relatedness were estimated, and phylogenetic tree drawing and population structure analysis were also performed. The results could aid in understanding the genomic inbreeding and relatedness of *H. bleekeri* individuals in Taibai River and potentially provide an opportunity to the genetic management of ex situ conservation and the proliferation and releasing to original habitats.

## 2 MATERIAL AND METHODS

### 2.1 Genetic samples of *Hucho bleekeri*

**Figure 1:**
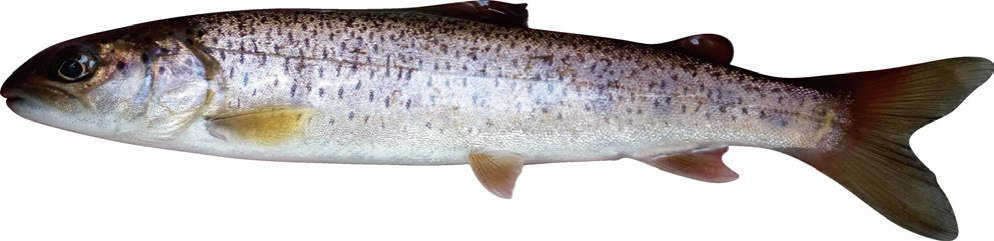
Yearling fish of *Hucho bleekeri* provided by Yongquan Zhang, with permission

**Figure 2:**
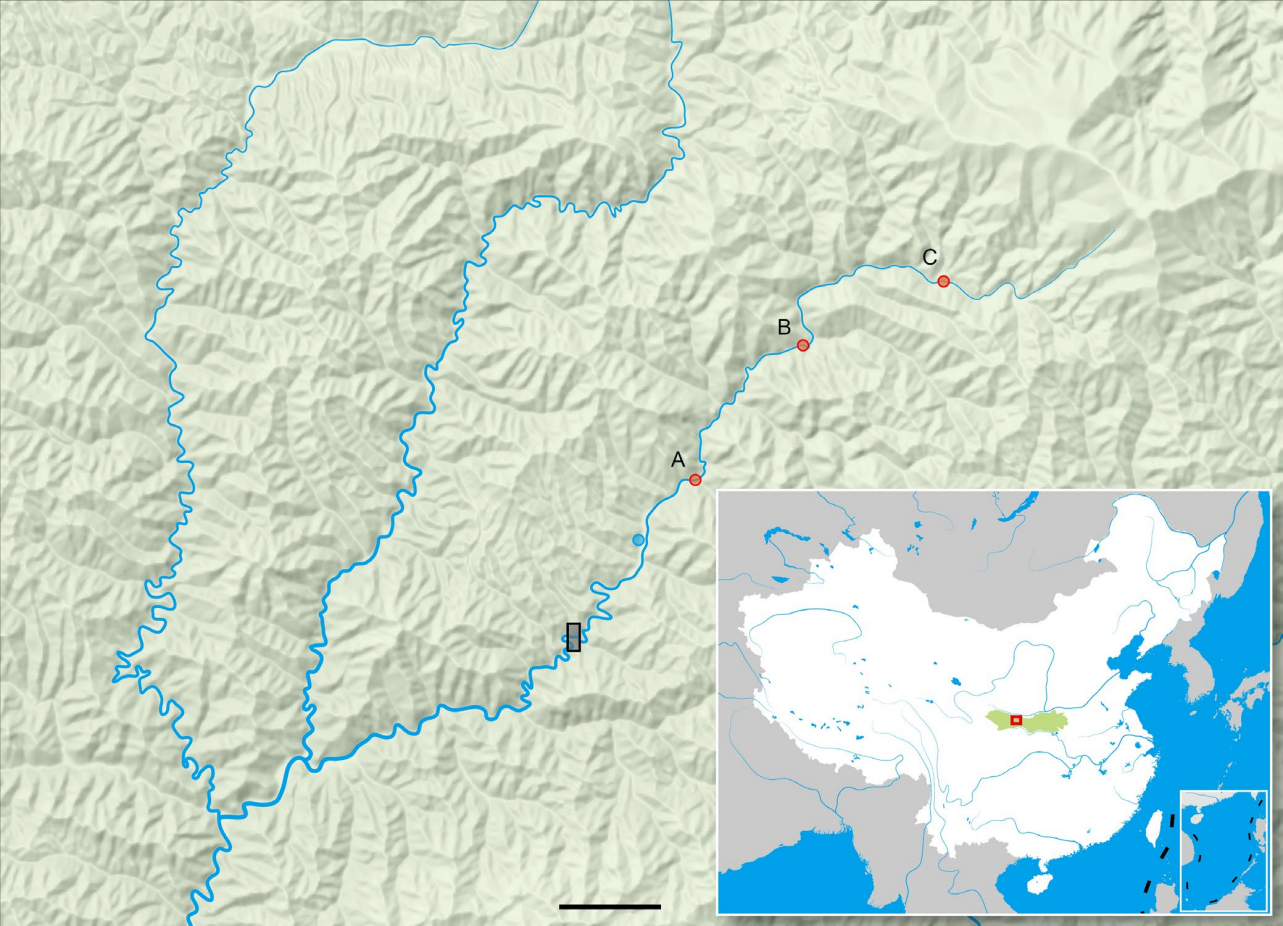
Three sampling locations for the 43 individuals of *Hucho bleekeri* collected in Taibai River. Blue circle represents Taibaihe town in Baoji City, Shaanxi Province, China. Each red circle represents a sampling location. The black rectangle represents a small hydropower station on Taibai River. Green block of the thumbnail map at lower right corner represents Qinling Mountains. Location A is about 5 km upstream from Taibaihe town. Location B is about 17 km upstream from Taibaihe town. Location C is about 25 km upstream from Taibaihe town. The small hydropower station is about 10 km downstream from Taibaihe town

Taibai River is a tributary of the Bao River and then flows into the Han River (the largest Yangtze River tributary). The river is 63 km long, with a drainage area of 1,349 km^2^ and an average annual flow of 17 m^3^/s, and the natural drop is 1,588 m (Wang, Wang, Luo, 1997). The sample with 43 yearling *H. bleekeri* (Figure 1) was collected from the Taibai River on July 2018. Taibai River has been sequestered from its access to the Bao River for more than 30 years by a hydropower dam without fish passage since late 1980s. The Taibaihe Town is affiliated to Taibai County, Baoji City, Shaanxi Province, and it is located in the southwest of Taibai County and is named after the Taibai River. It is between 107°03′ and 107°46′40″ east longitude, 33°38′13″ and 34°09′55″ north latitude, 107 km away from Taibai County. Due to inconvenient transportation, samples can only be collected along the river on foot. Therefore, three easy‐to‐reach sampling locations were selected in the upper reaches of Taibaihe Town, including location A (about 5 km upstream of Taibaihe Town), location B (about 17 km upstream of Taibaihe town), and location C (about 25 km upstream of Taibaihe town), and the hydropower dam is located about 10 kilometers downstream of Taibaihe Town (Schematic diagram of sampling locations shown in Figure 2).

Samples were collected with standard gillnetting procedures from Taibai River and were released immediately after taking part of the adipose fin. The number of samples in three locations was 11 individuals from location A, 20 individuals from location B, and 12 individuals from location C, respectively. The 2‐phenoxyethanol (C_8_H_10_O_2_) was used as an anesthetic to prevent injury to the fish during handling and cutting for some caudal fins. Then, fin samples were stored in absolute ethanol in a refrigerator at −20°C until the extraction of genomic DNA. All of the procedures using animals were conducted according to the guidelines for the care and use of experimental animals established by the Ministry of Science and Technology of the People's Republic of China (Document Number: 2006‐398) and were approved by the Academic Ethics Committee of Heilongjiang River Fisheries Research Institute of Chinese Academy of Fishery Sciences.

### 2.2 Genotyping of the *Hucho bleekeri* individuals

The total genomic DNA of *H. bleekeri* was extracted using a standard phenol–chloroform protocol, and DNA quality was checked by NanoDrop 2000C spectrophotometer (Thermo Scientific) and used for library construction. Considering the quality control criteria required for sequencing (32 μl genomic DNA solution content ≥5 μg DNA), all of the 43 individuals passed the criteria and sequenced using high‐throughput SLAF sequencing methodology. Fragments of genomic DNA of different lengths digested by the restriction enzymes were simulated in silico, and to predict the availability of 239,032 SLAF tags (>200,000), the restriction enzymes *Rsa*I and *Hae*III were used for genomic digestion based on SLAF alignments to the reference genome sequence of *Hucho hucho* (Genome accession number: ASM331708v1, released by University Of Aberdeen on July 2018). The clustering of the index‐coded samples was performed on a cBot Cluster Generation System using a TruSeq SR Cluster Kit v3‐cBot‐HS (Illumina Inc.), according to the manufacturers' instructions. After cluster generation, the library preparations were sequenced, and the 126 bp read at both ends of each 414–464 bp fragment was sequenced on an Illumina HiSeq ×10 platform Genome Analyzer (Illumina Inc.), according to the manufacturer's recommendations at the Experimental Department of BioMarker Technologies Corporation.

The development of SNP markers was performed with the *H. hucho* genome as a reference genome, too. The SLAF‐seq reads were aligned to the reference genome using BWA software (Li & Durbin, 2010), and the SNPs were developed via both GATK and SAMtools software (Li et al., 2009; McKenna et al., 2010), and SNPs discovered by both software were used for genetic analysis. The SNP marker intersection was used as the final reliable SNP marker dataset. Vcftools (Danecek et al., 2011) was used to filter SNPs meeting all four criteria: QUAL value ≥30, minor allele frequency (MAF) ≥5%, the significance level of Hardy–Weinberg equilibrium (HWE) test ≥0.01, and there were no more than 4 individuals with missing genotypes at each locus among all the 43 individuals.

### 2.3 Genetic model for estimating genomic relatedness

The genomic estimates of additive and dominance relatedness were obtained using a mixed model based on the quantitative genetics model (Falconer & Mackay, 1996) that partitions a genotypic value into its breeding value and dominance deviation (Da et al., 2014). Under the assumption of Hardy–Weinberg equilibrium (HWE), the mixed model to implement the traditional quantitative genetics model be expressed as:

(1) $$y=X\mu +Za+Zd+e=X\mu +ZM_{\alpha }\alpha +ZM_{\delta }\delta +e$$

where *μ* was fixed effect, *a* = *M*_α_ *α* was genomic breeding value and *d* = *M*_δ_ *δ* genomic dominance deviations, α was gene additive effects of SNPs, and δ was dominance effects of SNPs. The variance matrices were as follows: $\text{VAR}\left(a\right)=\sigma_{\alpha }^{2}A_{g}=\sigma_{\alpha }^{2}M_{\alpha }M_{\alpha }^{T}$, $\text{VAR}\left(d\right)=\sigma_{\delta }^{2}D_{g}=\sigma_{\delta }^{2}M_{\delta }M_{\delta }^{T}$, where $\sigma_{\alpha }^{2}$ was additive variance, and $\sigma_{\delta }^{2}$ was dominance variance. *A*_g_ was genomic additive relationship matrix, and *D*_g_ was genomic dominance relationship matrix.

Genomic relatedness between individuals can be described by genomic pairwise coancestry coefficients (*F_ij_* = *θ*) and genomic pairwise dominance coefficient (*D_ij_*). The genomic coancestry coefficient (*θ*) between individuals *i* and *j* was estimated based on the half off‐diagonal value in *A*_g_ and dominance coefficient (*D_ij_*) between individuals *i* and *j* based on the off‐diagonal value in *D*_g_. Additional methods of genomic relatedness and similarity were selected as follows: genomic identity by descent (IBD*_ij_*) to indicate two homologous alleles from a common ancestor and genomic identity by state (IBS*_ij_*) to the server as a measure of common alleles shared by two individuals.

Multidimensional scaling (MDS) was analyzed to obtain genomic relationships between individuals. MDS is a method similar to principal component analysis (PCA), and the MDS and PCA dimensions are highly correlated and should generate very similar 3D result views. The reason for using the MDS method instead of the PCA method in this study is that these two methods have different strategies for data compression processing (Garbe, Prakapenka, Tan, & Da, 2016). The MDS method uses the largest number of individuals and the SNPs shared among individuals. In contrast, the PCA method uses the largest number of SNPs and filters some individuals with large number of missing SNPs. PCA method is used more widely when the sample size is large. In the present study, there are only 43 individuals, and all the individuals should be used in the genetic analyses. Therefore, MDS method is more suitable than PCA for this study.

*A*_g_ and *D*_g_ were calculated using a definition of genomic correlation (Wang & Da, 2014), which was a within‐SNP standardization of each SNP using its own SNP variance, being the Definition VI implemented by GVCBLUP program (Wang et al., 2014). The genomic inbreeding coefficients of individuals (*f_i_* = *F*), IBD, IBS, and MDS were calculated by using PLINK software (Purcell et al., 2007).

### 2.4 Phylogenetic and genetic structure analysis

A maximum likelihood (ML) tree was constructed using the genome‐wide SNP markers with the MEGA X software (Kumar, Stecher, Li, Knyaz, & Tamura, 2018) with the Tamura–Nei model (Tamura & Nei, 1993), and the node support for ML trees was assessed with 1,000 bootstrap pseudoreplicates (Felsenstein, 1985). The genetic structure of the 43 individuals was analyzed with the genome‐wide SNP markers using ADMIXTURE software version 1.3 (Alexander, Novembre, & Lange, 2009). Prior population information was analyzed with genotype frequencies, using a number of clusters (*K*) ranging from 1 to 10, and ADMIXTURE's cross‐validation was used to choose the correct value for *K*.

### 2.5 The differences of genomic relatedness between individuals from the three sampling locations

The differences between *H. bleekeri* populations in genomic inbreeding coefficients, additive and dominance relationships, IBD, and IBS were tested using the R software. The statistical model was *y* = *μ* + population + *e*, where *y* = the observation, *μ* = common mean, population = effect of a population from the three sampling locations, and *e* = random residual. Three populations of A, B, and C were included in the statistical model for genomic inbreeding coefficients.

## 3 RESULTS

### 3.1 Statistics and evaluation of sequencing data

A total of 260.78 M paired‐end reads were generated for 43 fish by SLAF sequencing, and the average Q30 bases ratio was 94.85%, and the average GC content was 43.13%. After low‐quality reads, adaptors, and barcode sequences were removed, a total of 1,216,579 SLAF tags were obtained. Of the total SLAF tags, 220,703 were polymorphic and the average sequencing depth was 11.88‐fold. A total of 468,425 SNPs markers were obtained, among which there were 15,396 SNPs passed the SNP filtration criteria in this study.

### 3.2 Genomic inbreeding level estimates

**Figure 3:**
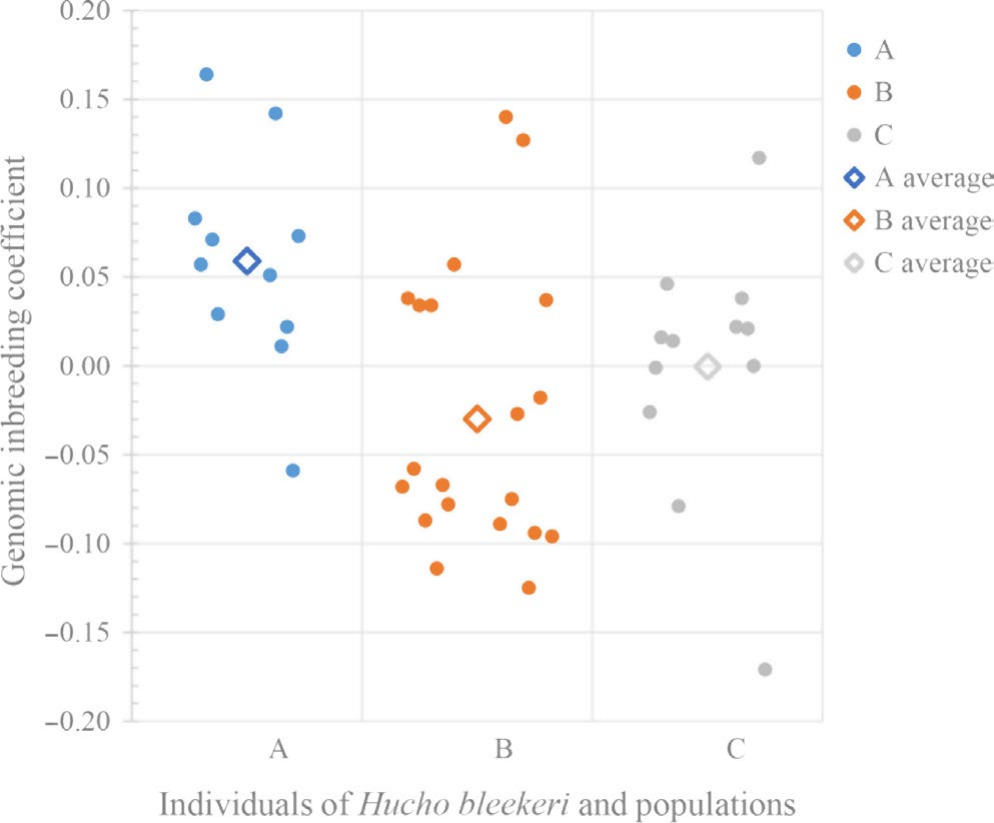
Genomic inbreeding coefficients of the 43 sequenced individuals and average genomic inbreeding coefficients of three populations

The average genomic inbreeding coefficients ($\bar{F}$) showed that the inbreeding level of this whole isolated population is at a low level ($\bar{F}$ = 2.6 × 10^−3^ ± 0.079), and population A had the highest genomic inbreeding coefficients, with average inbreeding coefficient of $\bar{F}$ = 0.058 ± 0.061 (Table 1), followed by C and B populations, with negative value close to zero (Table 1, Figure 3). Only the A population had significantly higher $\bar{F}$ than the B population (*p* < .05). No significant differences were observed between other two pairs, neither A and C, nor B and C (Table 1).

**Table 1:** Average genomic inbreeding coefficients by population

Population	Sample size	Genomic inbreeding coefficients (Mean ± *SD*)	Min	Max
A	11	0.058^a^ ± 0.061	−0.059	0.164
B	20	−0.026^b^ ± 0.079	−0.125	0.140
C	12	−0.00025^ab^ ± 0.071	−0.171	0.117
Total	43	0.0026 ± 0.079	−0.171	0.164

Note

Genomic inbreeding coefficients were analyzed using 15,396 SNP set by PLINK. A is the population of location A. B is the population of location B. C is the population of location C.

Within a row with no common lowercase superscript (a, b) differ significantly in multiple comparisons (*p* < .05).

In addition, due to the fact that the inbreeding in one individual is equal to the coancestry between parents, some individuals have inbreeding close to the expectation of mating among first cousins (0.0625, 3 in A population) and half‐sibs (0.125, 2 in A population, 2 in B population, and 1 in C population), indicating a mating among relatives of 45.5% in A (5/11), 10% in B (2/20), and 8.3% in C (1/12) populations, mean of 18.6% (8/43).

### 3.3 Genomic pairwise relatedness and similarity estimates within and between populations

**Figure 4:**
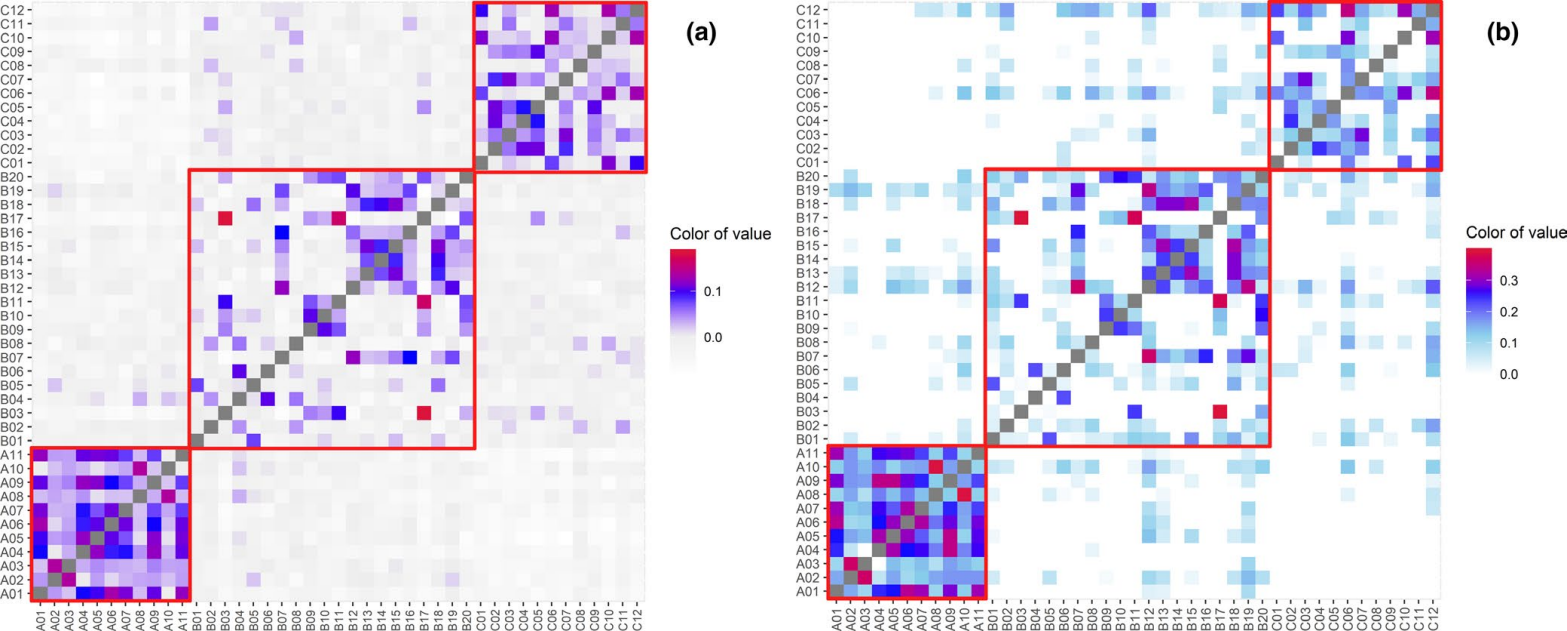
The overview diagram of genomic pairwise coancestry coefficients and probability of identical by descent (IBD) between the 43 sequenced individuals. (a) genomic pairwise coancestry coefficients. (b) Probability of alleles identical by descent (IBD). Red squares represent genomic pairwise coancestry coefficients and probability of IBD within each of three populations

**Figure 5:**
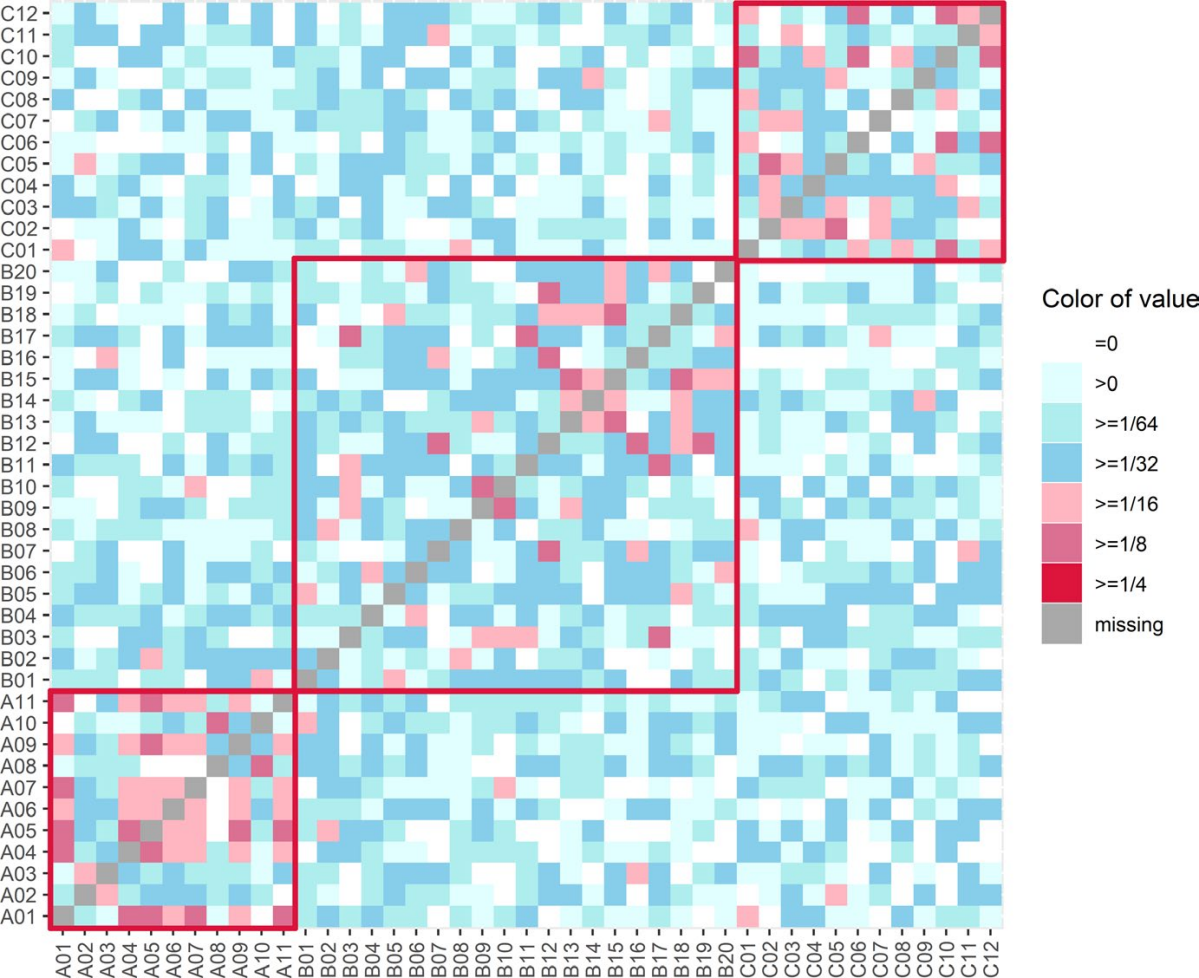
The overview diagram of genomic pairwise dominance coefficients between the 43 sequenced individuals. Red squares represent genomic pairwise dominance coefficients within each of three populations

**Figure 6:**
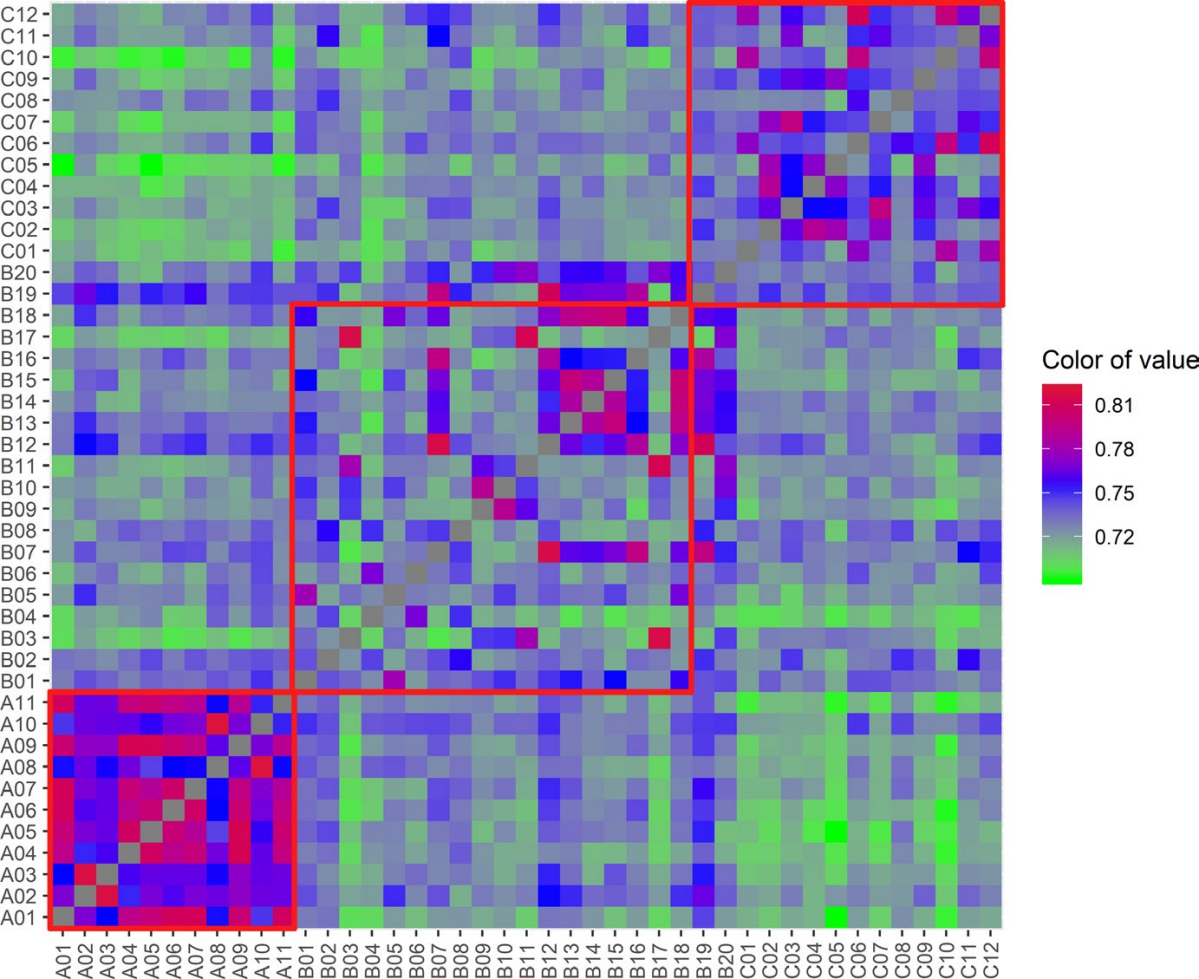
The overview diagram of probability of identical by state (IBS) between the 43 sequenced individuals. Red squares represent probability of IBS within each of three populations

Genomic pairwise relatedness and similarity of individuals pairs within or between populations from the three sampling locations can be identified by *θ*, *D*, IBD, and IBS. Individual pairs with a close genetic relationship can be validated by the consistency of four parameters. The graphical display for estimates of the genomic pairwise *θ*, *D*, IBD, and IBS analyses was shown in Figure 4 (for *θ* and IBD), Figure 5 (for *D*), and Figure 6 (for IBS), and the average genomic pairwise relatedness and similarity coefficient of above four parameters within each of three populations were shown in Table 2. Individual pairs within population A had the highest mean genomic relatedness and similarity, and individual pairs out of B and C populations had relatively distant genomic relatedness and similarity (Table 2). There were no individual pairs with a dominance coefficient ≥0.25, and some individual pairs exceeded 0.0625. The results of significance tests showed that the population A was very significantly different from the population B for all the four parameters of the *θ*, IBD, *D*, and IBS (*p* < .05). At the same time, population A was significantly different from the population C for all the four parameters, including *θ*, IBD, *D*, and IBS (*p* < .05). The difference between the populations B and C was significant for the *θ* and IBS (*p* < .05). Meanwhile, it was insignificant for the *D* and IBD between the two populations (*p* > .05; Table 2).

**Table 2:** Average genomic pairwise relatedness and similarity coefficient within each of three populations

Within population	N of pairs	*θ* (Mean ± *SD*)	IBD (Mean ± *SD*)	*D* (Mean ± *SD*)	IBS (Mean ± *SD*)
A	55	0.061^a^ ± 0.041	0.202^a^ ± 0.087	0.062^a^ ± 0.052	0.779^a^ ± 0.022
B	190	0.001^b^ ± 0.046	0.07^b^ ± 0.095	0.035^b^ ± 0.036	0.739^b^ ± 0.025
C	66	0.032^c^ ± 0.042	0.09^b^ ± 0.094	0.043^b^ ± 0.045	0.749^c^ ± 0.023

Note

*θ* and *D* were calculated using 15,396 SNPs set by GVCBLUP. IBD and IBS were calculated using 15,396 SNPs set by PLINK. A represents all pairs of individuals within population A; B represents all pairs of individuals within population B; C represents all pairs of individuals within population C.

Within a row with no common lowercase superscript (a, b, c) differ significantly in multiple comparisons (*p* < .05).

Abbreviations: *D*, genomic pairwise dominance coefficient; IBD, probability of identify by descent; IBS, probability of identify by state; *θ*, genomic pairwise coancestry coefficients.

**Figure 7:**
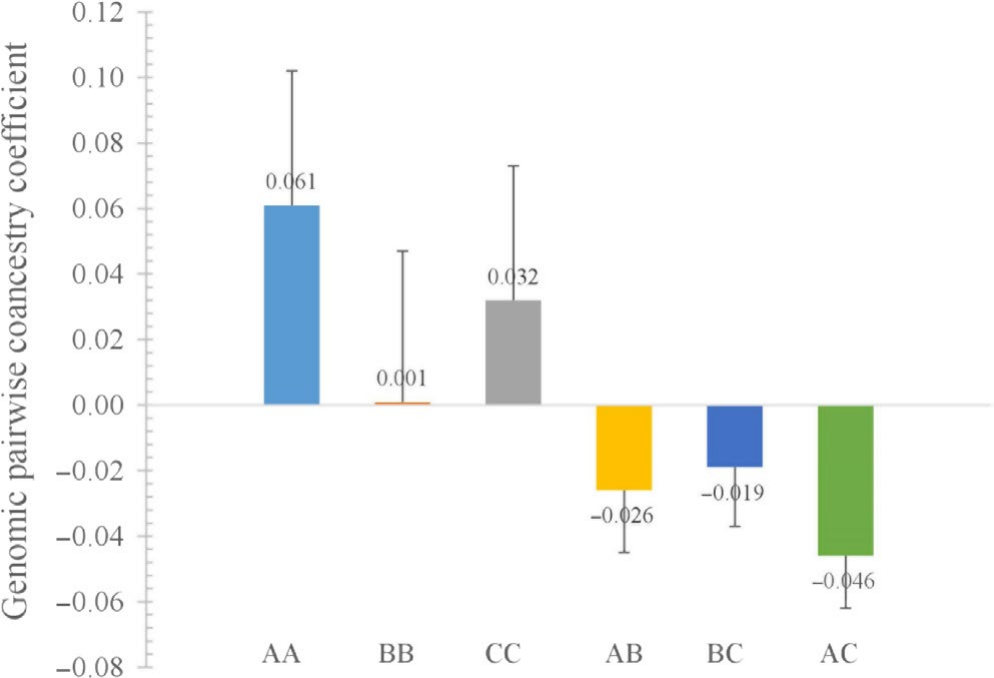
Average genomic pairwise coancestry coefficients within each of three populations and between different populations. AA, BB, and CC represent average genomic pairwise coancestry coefficients within populations. AB, BC, and AC represent average genomic pairwise coancestry coefficients between populations

**Figure 8:**
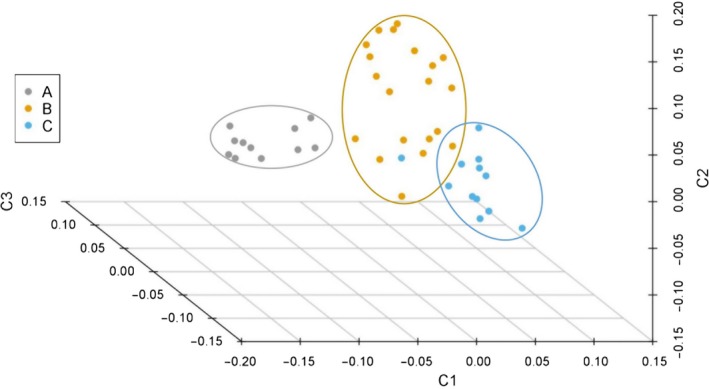
Three‐dimensional plot of Multidimensional Scaling (MDS) of 43 sequenced individuals. C1, C2, and C3 are the first three MDS dimensions of IBS distances analyzed by PLINK

**Figure 9:**
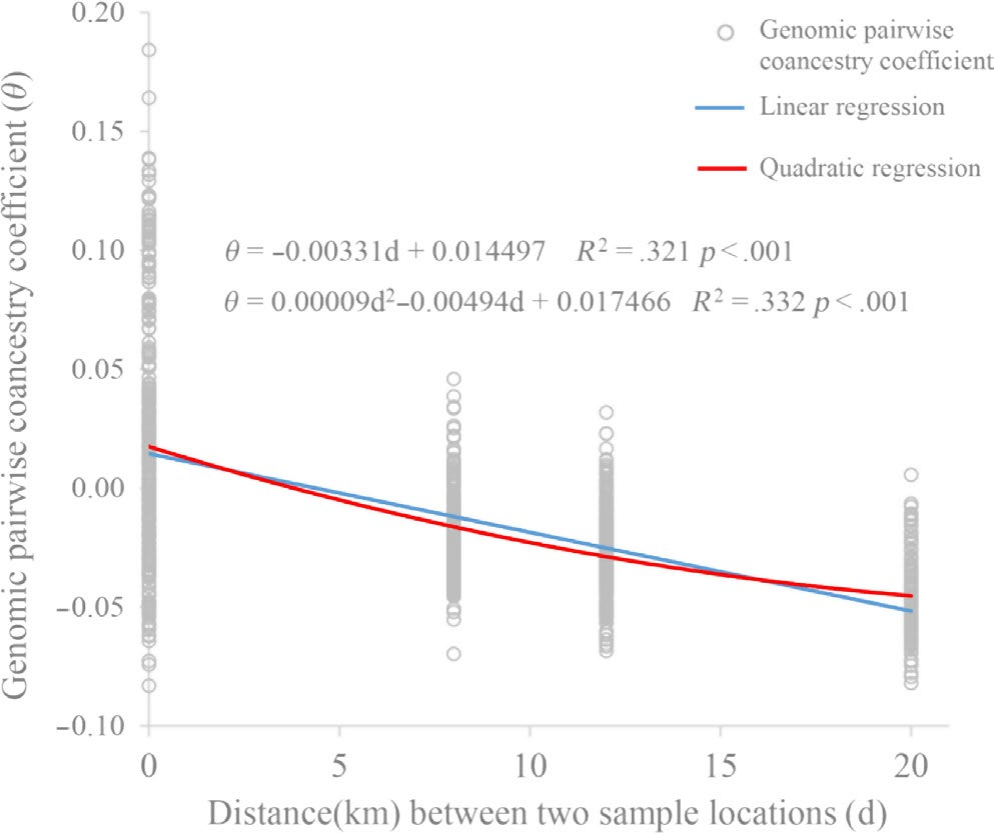
Regression analysis of genomic pairwise coancestry coefficients on the distance between two individuals

The means of genomic coancestry coefficient between different populations had negative values, with −0.019 for between populations B and C, −0.046 for between populations A and C, and −0.026 for between populations A and B (Table 3). Means of *θ* within each population were higher than the means between any two populations from the three sampling locations (Figure 7). A three‐dimensional view of the first three MDS components for the 43 individuals was presented in Figure 8, population A was separate from populations B and C, and the individuals from B and C populations cannot be distinguished independently. The result of regression analysis showed that *θ* was negatively correlated with the geographical distances between the sampling locations (*p* < .001; Figure 9).

**Table 3:** Average genomic pairwise relatedness and similarity coefficient between different populations

Between different populations	N of pairs	*θ* (Mean ± *SD*)	IBD (Mean ± *SD*)	*D* (Mean ± *SD*)	IBS (Mean ± *SD*)
A‐B	220	−0.026^a^ ± 0.019	0.015^a^ ± 0.032	0.020^a^ ± 0.021	0.728^a^ ± 0.013
A‐C	132	−0.046^b^ ± 0.016	0.004^b^ ± 0.018	0.016^a^ ± 0.022	0.715^b^ ± 0.012
B‐C	240	−0.019^c^ ± 0.018	0.020^a^ ± 0.041	0.019^a^ ± 0.020	0.726^a^ ± 0.011

Note

A‐B represents all pairs between individuals of population A and individuals of population B. A‐C represents all pairs between individuals of population A and individuals of population C. B‐C represents all pairs between individuals of population B and individuals of population C.

Within a row with no common lowercase superscript (a, b, c) differ significantly in multiple comparisons (*p* < .05).

Abbreviations: *D*, genomic pairwise dominance coefficient; IBD, probability of identify by descent; IBS, probability of identify by state; *θ*, genomic pairwise coancestry coefficients.

### 3.4 Phylogenetic tree and genetic structure mapping

**Figure 10:**
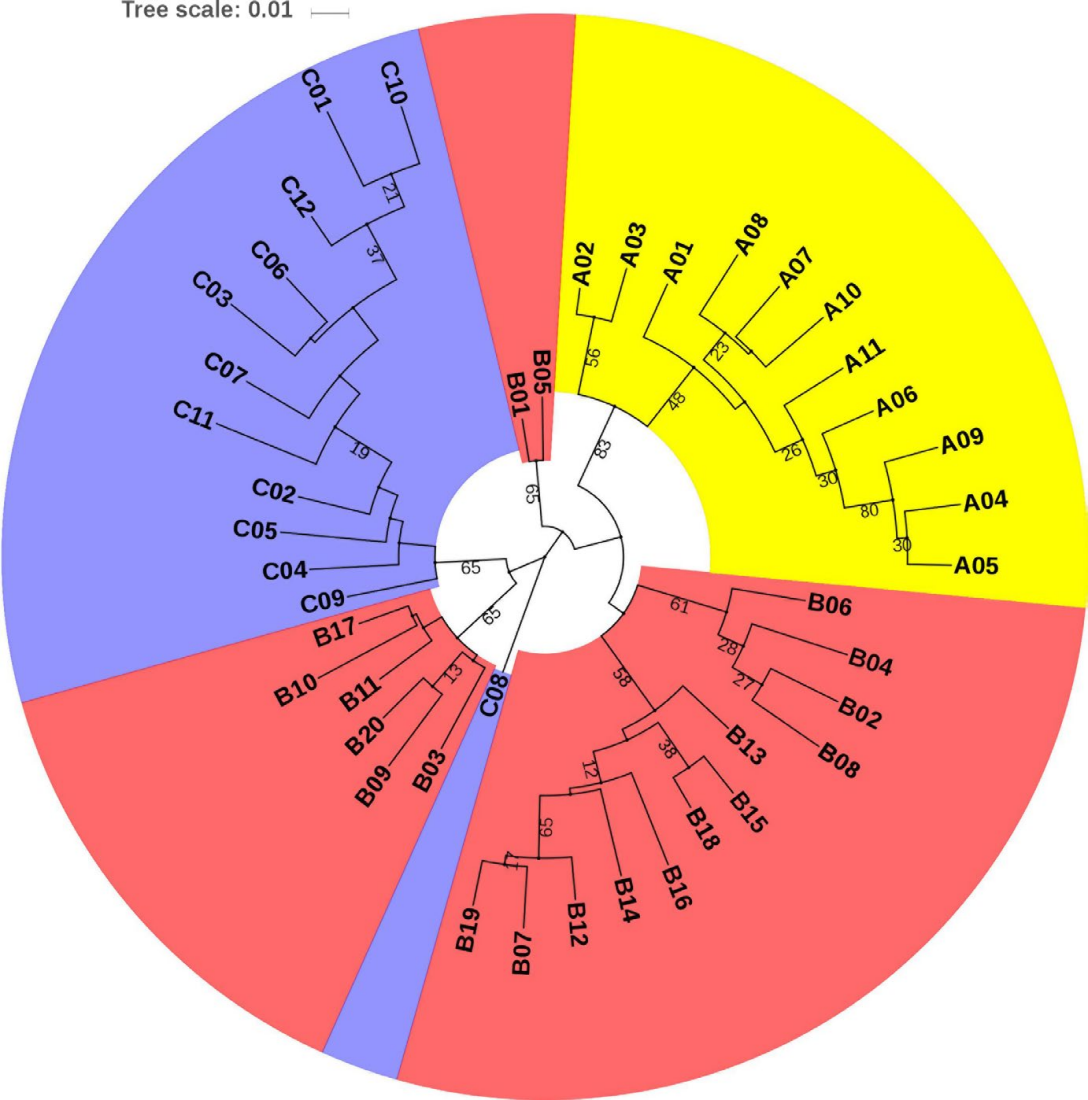
Maximum likelihood phylogenetic tree for the 43 sequenced individuals based on multiple sequence alignment of 15,396 SNPs. The yellow, pink, and purple blocks represent a population of three sampling locations respectively

**Figure 11:**
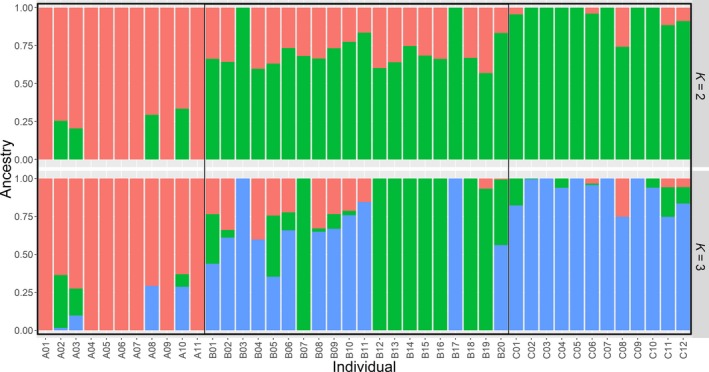
Genetic structure of the 43 sequenced individuals based on *K* = 2 and *K* = 3 using ADMIXTURE. Each column represents a single individual, and the proportion of the colored part represents the estimated genetic ancestry per individual

**Figure 12:**
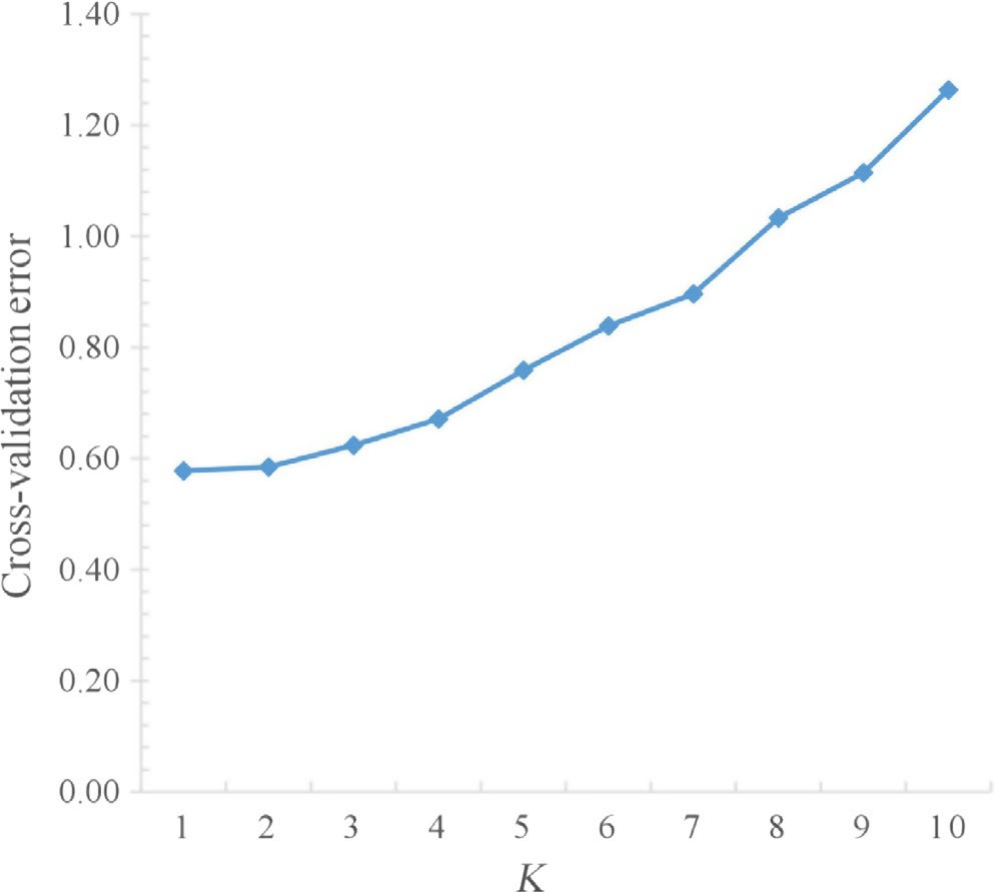
ADMIXTURE's cross‐validation plot of genetic structure analysis from *K* = 1–10

All the 15,396 SNPs were used to construct maximum likelihood (ML) phylogenetic tree with MEGA X and genetic structure with ADMIXTURE version 1.3 for the 43 individuals. Among the branches in the ML phylogenetic tree, all 11 individuals in population A were completely clustered in one branch, and neither individuals in population B or population C can be clustered together into a branch (Figure 10). Analysis of the genetic structure of 43 individuals revealed that the whole population can be considered to have two subpopulations (Figure 11), and cross‐validation results suggested *K* = 1 or 2 with the lowest probability of errors (Figure 12).

## 4 DISCUSSION

In the current densely populated areas such as East Asia, human activities are widely involved in various ecological types and many remote areas. Genetic diversity detection and management for fragmented animal populations are crucial importance in conservation biology (Frankham et al., 2017). Freshwater fishes are particularly vulnerable to loss genetic diversity because they often live in small, fragmented populations that are susceptible to habitat degradation, pollution, and the introduction of foreign species (Closs, Angermeier, Darwall, & Balcombe, 2015). *Hucho bleekeri* wild populations are severely threatened by habitat loss and illegal fishing, resulting in a “critically endangered” status in the IUCN Red List. The *H. bleekeri* sample in this study is from a typical isolated population living in Taibai River blocked by a hydropower station without a fish passage since late 1980s. In addition, the poor traffic conditions of Taibai River made it very difficult to obtain the fish sample, and we had to walk along the river bank to the sampling locations. Standard gillnetting procedures were used to catch yearling juvenile fish at three sampling locations, which took nearly 1 month and finally captured 43 individuals. Using the same sample size as Wang et al. (2016), we obtained the similar population genetic structure detected by using genome‐wide SNP markers as their result by microsatellite markers. Thus, we tend to believe the samples in this study were random enough and could be representative of the true population.

The results of genomic inbreeding and similarity measures provided estimates of actual levels of inbreeding and relatedness of *H. bleekeri* in Taibai River habitats and may be helpful to the species conservation including captive breeding and reintroduction programs. No obvious evidence of inbreeding (*F*_ST_ = 0.04041 and *F*_IS_ < 0) was detected using molecular variance constructed by 15 microsatellite markers for 43 individuals of *H. bleekeri* from the Taibai River in a previous contribution of other researchers (Wang et al., 2016). In this study, we directly estimated the genomic inbreeding coefficients using genome‐wide SNP markers. As a result, the low level of genomic inbreeding coefficient of the population ($\bar{F}$=2.6 × 10^−3^ ± 0.079) indicated a relatively optimistic inbreeding status for this isolated wild fish population. This result along with the microsatellite result (Wang et al., 2016) suggested many unrelated breeding individual's mates in this isolated *H. bleekeri* population living in the Taibai River. At the same time, we also found inbreeding evidence of mating between close relatives in this small population, and 3 (6.98% of 43) individuals were close to the expectation of mating among first cousins (*F* = 0.0625) and 5 (11.63% of 43) from mating among half‐sibs (*F* = 0.125).

According to classical quantitative genetics theory, only full‐sibs are expected to have nonzero dominance coefficient with the expected value of *D* = 0.25 (1/4) (Falconer & Mackay, 1996). Other than this, the other type nonzero dominance relationship is double first cousin with expected dominance relationship of *D* = 0.0625 (1/16). In the present study, there were no individual pairs with a dominance coefficient ≥0.25, but some individual pairs exceeded 0.0625 (Charmantier, Garant, & Kruuk, 2014). The extensive and low dominance coefficient between individual pairs indicated that there were no full siblings among the captured 43 yearling individuals. This result suggested that at least 43 female broodstocks were involved in reproduction in the Taibai River. Considering the tens of thousands of eggs produced by the fish, it also implied that the survival rate of each family's offspring was very low caused by reasons that had not been quantified.

The low value of genomic coancestry coefficient and IBD for interpopulations, along with MDS and ML phylogenetic tree, results visually displayed population A was relatively genetic independent from the populations B and C in Taibai River. Meanwhile, negative correlation between coancestry coefficient and geographical distances was observed for the three sampling locations connected to each other in the same river suggested that the swimming ability of juvenile *H. bleekeri* living in Taibai River did not seem to be able to reach a relatively wide range, and long distance may be effective in blocking gene flow between individuals. Similar to the findings in giant pandas (Garbe et al., 2016), the genetically unrelated individuals and potentially unrelated spawning locations would provide an opportunity to use spawning‐location‐controlled individuals sharing no IBD alleles as mating candidates for species conservation to avoid inbreeding. This strategy of controlling mating by individuals from different unrelated geographical spawning locations would minimize the probability of hidden relatedness between parents used for conservation. At the same time, this study provided a genomic‐relatedness‐guided breeding and conservation strategy for wild fish species without pedigree information records. We can directly control the individuals involved in mating by setting the index of genomic inbreeding and similarity measures. In fact, irrelevant geographical spawning location‐directed mating and genomic relatedness information‐guided mating were equivalent in the case of all genome‐wide SNP markers that were available for all the individuals involved in mating.

Slightly negative genomic inbreeding coefficient and pairwise relatedness estimates within and between populations were common in this study. In particular, average genomic pairwise coancestry coefficients between different populations were negative. In general, these small negative estimates were considered as “unrelated” (Ackerman et al., 2017; Garbe et al., 2016). However, it also suggested that there may be some bias in the parameter estimation (Zhou, Vales, Wang, & Zhang, 2016). The reasons for this phenomenon were that the molecular markers obtained by the SLAF sequencing method were not uniformly covered on the genome, and higher‐density and coverage genomic molecular markers can be obtained using higher‐throughput sequencing methods such as resequencing to increase the statistical power (Ackerman et al., 2017; Da et al., 2014). Furthermore, including the focal individual in the estimation of the allele frequencies may be the cause of this bias (Ackerman et al., 2017), too. Nevertheless, the robustness of these estimates needs to be re‐evaluated when a better reference genome of *H. bleekeri* but not *H. hucho* becomes available.

In summary, the approach of genomic inbreeding and similarity measures allows the estimation of inbreeding and coancestry of the *H. bleekeri* without pedigree records. The low levels of genomic inbreeding and relatedness of the *H. bleekeri* individuals in Taibai River suggested a relatively large number of sexually mature individuals were involved in reproduction. Genomic differentiation between the three sampling locations was negatively correlated with the geographical distances, and the fish in location A could be considered as genetically independent from the other two locations. This study suggested a genomic‐relatedness‐guided breeding and conservation strategy for wild fish species without pedigree information records.

## CONFLICT OF INTEREST

None declared.

## AUTHOR CONTRIBUTIONS

GH and JY: conceived and designed the experiments. YZ collected samples. PL, YZ, GR, and GH performed the experiments. GH and PL analyzed the data. GH and PL wrote the paper.

## Data Availability

All the raw sequence data were submitted to BIG database (The BIG Data Center at Beijing Institute of Genomics) and are publicly available (Accession numbers: PRJCA001798). All descriptive statistics for the sequencing dataset, as well as genetic parameter estimates of all individuals, genomic relatedness, and similarity measures for each individual pair within and between three populations, were stored in Dryad (https://doi.org/10.5061/dryad.bg79cnp79).

## References

[ece35994-bib-0001] Ackerman, M. S. , Johri, P. , Spitze, K. , Xu, S. , Doak, T. G. , Young, K. , & Lynch, M. (2017). Estimating seven coefficients of pairwise relatedness using population‐genomic data. Genetics, 206, 105–118. 10.1534/genetics.116.190660 28341647 PMC5419463

[ece35994-bib-0002] Alexander, D. H. , Novembre, J. , & Lange, K. (2009). Fast model‐based estimation of ancestry in unrelated individuals. Genome Research, 19, 1655–1664. 10.1101/gr.094052.109 19648217 PMC2752134

[ece35994-bib-0003] Charmantier, A. , Garant, D. , & Kruuk, L. E. B. (2014). Quantitative genetics in the wild. Oxford, UK: Oxford University Press.

[ece35994-bib-0004] Closs, G. P. , Angermeier, P. L. , Darwall, W. R. T. , & Balcombe, S. R. (2015). Why are freshwater fish so threatened? In Closs G. P., Olden J. D. & Krkosek M. (Eds.), Conservation of freshwater fishes (pp. 37–75). Cambridge, UK: Cambridge University Press.

[ece35994-bib-0005] Da, Y. , Wang, C. , Wang, S. , & Hu, G. (2014). Mixed model methods for genomic prediction and variance component estimation of additive and dominance effects using SNP markers. PLoS One, 9, e87666 10.1371/journal.pone.0087666 24498162 PMC3907568

[ece35994-bib-0006] Danecek, P. , Auton, A. , Abecasis, G. , Albers, C. A. , Banks, E. , DePristo, M. A. , … Durbin, R. (2011). The variant call format and VCFtools. Bioinformatics, 27, 2156–2158. 10.1093/bioinformatics/btr330 21653522 PMC3137218

[ece35994-bib-0007] Du, H. , Li, L. , Wei, Q. , Zhang, S. , Wang, C. , Sun, Q. , … Li, L. (2014). The rediscovery of *Hucho bleekeri* in the Taibai River, the upper tributary of the Han River, China. Chinese Journal of Zoology, 3, 414 (In Chinese).

[ece35994-bib-0008] Falconer, D. S. , & Mackay, T. F. C. (1996). Introduction to quantitative genetics (4th ed.). London, UK: Longman.

[ece35994-bib-0009] Felsenstein, J. (1985). Confidence limits on phylogenies: An approach using the bootstrap. Evolution, 39, 783–791. 10.1111/j.1558-5646.1985.tb00420.x 28561359

[ece35994-bib-0010] Frankham, R. , Ballou, J. D. , Ralls, K. , Eldridge, M. D. B. , Dudash, M. R. , Fenster, C. B. , … Sunnucks, P. (2017). Genetic management of fragmented animal and plant populations. Oxford, UK: Oxford University Press.

[ece35994-bib-0011] Garbe, J. R. , Prakapenka, D. , Tan, C. , & Da, Y. (2016). Genomic inbreeding and relatedness in wild panda populations. PLoS One, 11, e0160496 10.1371/journal.pone.0160496 27494031 PMC4975500

[ece35994-bib-0012] Goddard, M. E. , Hayes, B. J. , & Meuwissen, T. (2011). Using the genomic relationship matrix to predict the accuracy of genomic selection. Journal of Animal Breeding and Genetics, 128, 409–421. 10.1111/j.1439-0388.2011.00964.x 22059574

[ece35994-bib-0013] Hu, M. , Wang, Y. , Cao, L. , & Xiong, B. (2008). Threatened fishes of the world: *Hucho bleekeri Kimura*, 1934 (Salmonidae). Environmental Biology of Fishes, 82, 385–386.

[ece35994-bib-0015] Kumar, S. , Stecher, G. , Li, M. , Knyaz, C. , & Tamura, K. (2018). MEGA X: Molecular evolutionary genetics analysis across computing platforms. Molecular Biology and Evolution, 35, 1547–1549. 10.1093/molbev/msy096 29722887 PMC5967553

[ece35994-bib-0016] Li, H. , & Durbin, R. (2010). Fast and accurate long‐read alignment with Burrows‐Wheeler transform. Bioinformatics, 26, 589–595. 10.1093/bioinformatics/btp698 20080505 PMC2828108

[ece35994-bib-0017] Li, R. , Yu, C. , Li, Y. , Lam, T. W. , Yiu, S. M. , Kristiansen, K. , & Wang, J. (2009). SOAP2: An improved ultrafast tool for short read alignment. Bioinformatics, 25, 1966–1967. 10.1093/bioinformatics/btp336 19497933

[ece35994-bib-0018] McKenna, A. , Hanna, M. , Banks, E. , Sivachenko, A. , Cibulskis, K. , Kernytsky, A. , … DePristo, M. A. (2010). The genome analysis toolkit: A MapReduce framework for analyzing next‐generation DNA sequencing data. Genome Research, 20, 1297–1303. 10.1101/gr.107524.110 20644199 PMC2928508

[ece35994-bib-0019] Meuwissen, T. , Hayes, B. , & Goddard, M. (2001). Prediction of total genetic value using genome‐wide dense marker maps. Genetics, 157, 1819–1829. 11290733 10.1093/genetics/157.4.1819 PMC1461589

[ece35994-bib-0020] Purcell, S. , Neale, B. , Todd‐Brown, K. , Thomas, L. , Ferreira, M. A. R. , Bender, D. , … Sham, P. C. (2007). PLINK: A tool set for whole‐genome association and population‐based linkage analyses. The American Journal of Human Genetics, 81, 559–575. 10.1086/519795 17701901 PMC1950838

[ece35994-bib-0021] Qi, D. , Chao, Y. , Yang, C. , Shen, Z. , & Tang, W. (2009). Cloning of mitochondrial cytochrome b gene of *Hucho bleekeri* and its phylogenetic relationship in subfamily Salmoninae. Sichuan Journal of Zoology, 28, 805–809. (In Chinese).

[ece35994-bib-0022] Shen, Z. , Tang, W. , & Li, K. (2006). The analysis of population dynamics of *Hucho bleeke*ri in Markehe River, Qinghai Province. Reserv Fisheries, 26, 71–73. (In Chinese).

[ece35994-bib-0023] Song, Z. (2012). *Hucho bleekeri*. The IUCN red list of threatened species. Version 2014.3.

[ece35994-bib-0024] Sun, X. , Liu, D. , Zhang, X. , Li, W. , Liu, H. , Hong, W. , … Zheng, H. (2013). SLAF‐seq: An efficient method of large‐scale de novo SNP discovery and genotyping using high‐throughput sequencing. PLoS One, 8, e58700 10.1371/journal.pone.0058700 23527008 PMC3602454

[ece35994-bib-0025] Tamura, K. , & Nei, M. (1993). Estimation of the number of nucleotide substitutions in the control region of mitochondrial DNA in humans and chimpanzees. Molecular Biology and Evolution, 10, 512–526. 8336541 10.1093/oxfordjournals.molbev.a040023

[ece35994-bib-0026] VanRaden, P. (2008). Efficient methods to compute genomic predictions. Journal of Dairy Science, 91, 4414–4423. 10.3168/jds.2007-0980 18946147

[ece35994-bib-0027] Wang, C. , & Da, Y. (2014). Quantitative genetics model as the unifying model for defining genomic relationship and inbreeding coefficient. PLoS One, 9, e114484 10.1371/journal.pone.0114484 25517971 PMC4269408

[ece35994-bib-0028] Wang, C. , Prakapenka, D. , Wang, S. , Pulugurta, S. , Runesha, H. B. , & Da, Y. (2014). GVCBLUP: A computer package for genomic prediction and variance component estimation of additive and dominance effects. BMC Bioinformatics, 15, 270 10.1186/1471-2105-15-270 25107495 PMC4133608

[ece35994-bib-0029] Wang, K. , Zhang, S. H. , Wang, D. Q. , Wu, J. M. , Wang, C. Y. , & Wei, Q. W. (2016). Conservation genetics assessment and phylogenetic relationships of critically endangered *Hucho bleekeri* in China. Journal of Applied Ichthyology, 32, 343–349.

[ece35994-bib-0030] Wang, K. , Zhang, S. , Wang, D. , Xin, M. , Wu, J. , Sun, Q. , … Wei, Q. (2015). Development of 27 novel cross‐species microsatellite markers for the endangered *Hucho bleekeri* using next‐generation sequencing technology. Conservation Genetics Resources, 7, 263–267. 10.1007/s12686-014-0353-y

[ece35994-bib-0031] Wang, Y. , Guo, R. , Li, H. , Zhang, X. , Du, J. , & Song, Z. (2011). The complete mitochondrial genome of the Sichuan taimen (*Hucho bleekeri*): Repetitive sequences in the control region and phylogenetic implications for Salmonidae. Marine Genomics, 4, 221–228. 10.1016/j.margen.2011.06.003 21867975

[ece35994-bib-0032] Wang, J. , Wang, B. , & Luo, Z. (1997). The Yangtze River Dictionary (In Chinese). Wuhan, China: Wuhan Publishing House.

[ece35994-bib-0033] Xu, W. , Sun, H. , Guan, H. , Kuang, Y. , Lu, J. , & Yin, J. (2007). Growth development and reproduction of reared *Hucho taimen* . Journal of Fishery Sciences of China, 14, 896–902 (In Chinese).

[ece35994-bib-0034] Zhang, S. , Wei, Q. , Du, H. , & Li, L. (2016). The complete mitochondrial genome of the endangered *Hucho bleekeri* (Salmonidae: Huchen). Mitochondrial DNA Part A, 27, 124–125. 10.3109/19401736.2013.878906 24617457

[ece35994-bib-0035] Zhou, Y. , Vales, M. I. , Wang, A. , & Zhang, Z. (2016). Systematic bias of correlation coefficient may explain negative accuracy of genomic prediction. Briefings in Bioinformatics, 18, 744–753. 10.1093/bib/bbw064 27436121

